# Machine learning–guided feature selection and predictive model construction for attention-deficit/hyperactivity disorder

**DOI:** 10.3389/fpsyt.2025.1724359

**Published:** 2025-12-17

**Authors:** Haojie Meng, Songtao Li, Xiwen Xing, Ruyi Fu, Yang Li, Qianqi Liu, Xu Wang

**Affiliations:** 1 Department of Children Health Care, Children’s Hospital of Nanjing Medical University, Nanjing, Jiangsu, China; 2 Department of Clinical Laboratory, Children’s Hospital of Nanjing Medical University, Nanjing, Jiangsu, China; 3 Department of Respiratory Medicine, Children’s Hospital of Nanjing Medical University, Nanjing, Jiangsu, China; 4 Department of Neurology, Children’s Hospital of Nanjing Medical University, Nanjing, Jiangsu, China; 5 Clinical Medical Research Center, Children’s Hospital of Nanjing Medical University, Nanjing, Jiangsu, China

**Keywords:** attention deficit/hyperactivity disorder, machine learning, routine blood counts, serum biochemical parameters, systemic inflammation markers

## Abstract

**Background:**

Attention Deficit/Hyperactivity Disorder (ADHD) is a highly prevalent neurodevelopmental disorder, but its diagnosis remains constrained. This study aimed to identify potential candidate indicators and construct an interpretable machine learning model for the identification of ADHD.

**Methods:**

A total of 8,598 children were enrolled and classified into three groups: ADHD (n=3,678), subthreshold ADHD (s-ADHD) (n=1,495), and healthy controls (HC) (n=3,425). Data collection covered 40 variables, including demographics, routine blood counts, serum biochemical parameters, body composition and systemic inflammation markers. Analysis of Variance (ANOVA) compared differences among the three groups, and key predictors were selected via Least Absolute Shrinkage and Selection Operator (LASSO) regression. Five machine learning models (Decision Tree, Random Forest, Multilayer Perceptron, Extreme Gradient Boosting, and Light Gradient Boosting Machine [LightGBM]) were developed for three clinically relevant binary classification tasks. SHapley Additive exPlanations (SHAP) values were applied to interpret the optimal model.

**Results:**

ANOVA indicated significant differences (*P* < 0.05) in most parameters among the three groups. However, *post-hoc* Least Significant Difference (LSD) tests showed that compared with HC, the ADHD group showed elevated inflammatory markers (NLR, PLR, SII), glucose, body mass index(BMI), and body fat percentage, but reduced albumin, total cholesterol, and lymphocyte counts. Similar alterations were observed in the s-ADHD group, showing a pattern consistent with that of the ADHD group. LASSO regression (λ.1se=0.038) selected 11 core predictors, with age, RDW-SD, sex, calcium, glucose, and albumin among the most contributing variables. Among the models, LightGBM demonstrated the best performance when distinguishing ADHD from HC (AUC = 0.924 with 36 features vs. AUC = 0.885 with 11 features). However, the model failed to effectively distinguish between ADHD and s-ADHD.

**Conclusions:**

This study reveals potential candidate indicators of ADHD and establishes an interpretable, low-cost machine learning model based on routine clinical data, offering a promising tool for early screening and clinical decision support.

## 1 Introduction

Attention Deficit/Hyperactivity Disorder (ADHD) is among the most prevalent childhood neurodevelopmental disorders globally, characterized by persistent symptoms of hyperactivity, impulsivity, and inattention (1). Epidemiological studies estimate the global prevalence of ADHD to be approximately 8% (2). In some cases, symptoms persist into adulthood (3), where individuals with ADHD are more likely to develop substance and alcohol use disorders and engage in antisocial behavior, and may also present, with anxiety and depressive disorders (4, 5). This poses serious challenges for public health and social governance. The etiology and pathophysiology of ADHD remain unclear, with converging evidence implicating genetic (6, 7), neurobiological, environmental (8, 9), and psychosocial factors.

In individuals with ADHD, neuroinflammation, such as leukocyte infiltration into the central nervous system and compromised blood-brain barrier integrity, have been implicated in the pathogenesis of ADHD (10). Systemic inflammation and immune dysregulation are increasingly recognized as potential contributors to ADHD pathophysiology (11, 12). Peripheral hematological markers, including neutrophil-to -lymphocyte ratio (NLR), platelet-to-lymphocyte ratio (PLR), red cell distribution width-to-platelet ratio (RPR), and systemic immune-inflammation index (SII), have been proposed as accessible indicators of inflammatory status (11, 12). Cholesterol is a key component of cell membranes, essential for maintaining fluidity, structural stability, and signal transduction (13). Evidence suggests an association between cholesterol and hyperactive behaviors (14), with low cholesterol levels linked to increased aggression and impulsivity. In addition, cholesterol metabolism plays an important role in both the induction and resolution of inflammatory responses (5).

Machine learning (ML) models are increasingly applied in medical research, offering powerful tools for analyzing complex datasets and enabling early disease detection and prognostic assessment across diverse fields such as oncology (15), medical imaging, neurology (16), cardiology (17), orthopedics, and infectious diseases. Handelman (18) have argued for ML as a critical tool for managing large heterogeneous datasets, emphasizing its potential to advance personalized medicine, computer-assisted diagnosis, and global health care improvement. In ADHD research, current applications have primarily centered on treatment prognosis (19), neuroimaging analyses (20) and retinal fundus imaging (21), with relatively few studies exploring biological or clinical associative markers derived from routine data.

Although increasing evidence has advanced understanding of ADHD pathophysiology, its diagnosis still relies primarily on behavioral assessments and clinical judgment, with limited objective laboratory support. Routine hematological and biochemical parameters can reflect systemic inflammation, immune status, and metabolic alterations, which are increasingly implicated in neurodevelopmental disorders. Readily available clinical indicators, including blood counts, biochemical indices, and body composition measures, are cost-effective and easily accessible. They may provide valuable clues to the underlying mechanisms of ADHD and hold promise as practical diagnostic associative markers. In this study, we included children with ADHD, children exhibiting inattentive, hyperactive or impulsive behaviors (subthreshold ADHD), and healthy controls to analyze laboratory indices and routine clinical parameters. Based on these data and Least Absolute Shrinkage and Selection Operator (LASSO) regression for feature selection, we developed machine learning models to enable early ADHD identification, offering a clinically feasible and cost-effective approach to support diagnostic.

## 2 Methods

### 2.1 Study design and participants

**Figure 1:**
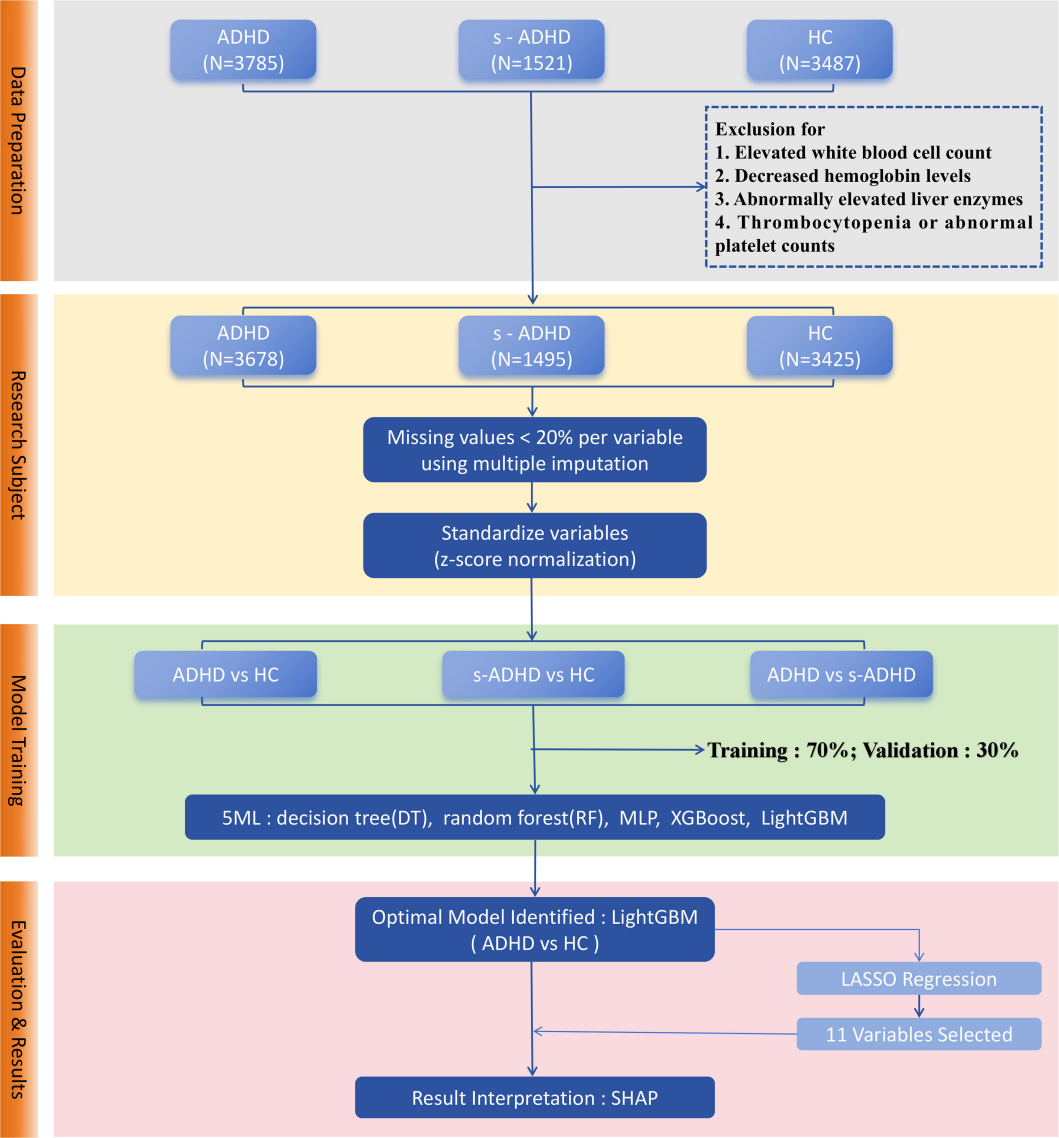
Workflow of machine learning model development for ADHD classification. Data preprocessing included outlier removal, random imputation, and z-score normalization, followed by a stratified random split with a 7:3 ratio. Five models (DT, RF, XGBoost, LightGBM, MLP) were trained for three pairwise classifications. Model performance was assessed using AUC, accuracy, sensitivity, specificity, and related metrics with ROC and PR curves. LightGBM achieved the best performance. LASSO regression selected 11 stable predictors, and SHAP analysis visualized feature contributions.

We conducted a cross-sectional study, analyzing outpatient records from the Children’s Hospital of Nanjing Medical University between January and November 2023. The study population included children diagnosed with ADHD, subthreshold ADHD cases and the healthy controls. ADHD was diagnosed according to the Diagnostic and Statistical Manual of Mental Disorders (22), Fifth Edition (DSM-5) (1). A behavioral rating scale derived from the DSM-5 symptom criteria was used, consisting of 18 items recorded in a binary (met/not met) format and divided into two domains, including inattention and hyperactivity/impulsivity. Children who met six or more criteria in either domain, with symptoms lasting at least six months and resulting in clinically significant impairment in social or academic functioning, were diagnosed with ADHD. Subthreshold ADHD (s-ADHD) cases were defined as children who exhibited behavioral manifestations of inattention, hyperactivity/impulsivity at home or school but met fewer than six criteria in either domain, and therefore did not fulfill the full DSM-5 diagnostic criteria. Healthy controls (HC) were children who underwent routine health check-ups during the same period and had no symptoms of ADHD. Children in the acute phase of infection, or with platelet abnormalities, reduced hemoglobin levels, or elevated liver enzyme concentrations were excluded from the study (**Figure 1**). Ultimately, a total of 8,598 children were included in the study, comprising 3,678 confirmed ADHD cases, 1,495 s-ADHD, and 3,425 HC.

This study was approved by the Ethics Committee of Nanjing Children’s Hospital. In accordance with institutional and ethical guidelines, informed consent was not required, as this retrospective study did not involve the collection of identifiable patient information.

### 2.2 Laboratory measurements

Demographic characteristics, routine blood counts, serum biochemical parameters, and body composition data were collected for all participants. Venous blood samples were analyzed for routine blood counts according to the WS/T 779–2021 industry standard and for serum biochemical parameters using the Roche automated platform. Biochemical and routine blood counts were collected for all participants, with detailed descriptions provided in **Supplementary Table S1**. Body composition was assessed with the InBody system (version LookinBody120.4.0.0.6), focusing primarily on body mass index (BMI) and percentage body fat (PBF). Age and sex were included as demographic covariates. In addition, several composite indices commonly regarded as markers of systemic inflammation (23) were calculated, including the neutrophil-to-lymphocyte ratio (NLR, neutrophil count/lymphocyte count), platelet-to-lymphocyte ratio (PLR, platelet count/lymphocyte count), red cell distribution width-to-platelet ratio (RPR, RDW-CV/platelet count), and systemic immune-inflammation index (SII, platelet count × neutrophil count/lymphocyte count).

### 2.3 Statistical analysis

All statistical analyses were performed using R (version 4.2.2), and python (version 3.12). Continuous variables were expressed as mean ± standard deviation (SD), and categorical variables as counts and percentages (%). Normality of continuous variables was assessed using scatter plots, and missing values due to random loss were imputed using multiple imputation (24). Differences among ADHD, s-ADHD, and HC groups were assessed using one-way Analysis of Variance (ANOVA), followed by *post-hoc* comparisons using the Least Significant Difference (LSD) test. A two-tailed *P* < 0.05 was considered statistically significant.

### 2.4 Machine learning methods

**Figure 1** shows a technical flowchart of the four core steps: data preparation, research subject screening, model training, and evaluation & results, which clearly presents the machine learning analysis workflow of this study. We applied five machine learning methods: Decision Tree(DT), Random Forest(RF), Multilayer Perceptron (MLP), Extreme Gradient Boosting (XGBoost), and Light Gradient Boosting Machine (LightGBM) (25). Derived inflammatory ratios (NLR, PLR, RPR, SII) were excluded from the machine learning models to avoid redundancy and multicollinearity with their component variables. All other biochemical indices, routine blood counts, demographic characteristics and body composition indices were included in the models. DT (26) is a non-parametric supervised learning method that makes predictions by recursively partitioning the feature space into regions. RF (27) is an ensemble learning algorithm that combines multiple decision trees using bootstrap aggregation to reduce overfitting, enhance generalization. XGBoost, an efficient implementation of gradient boosting, was configured with logistic loss and regularization to enhance predictive accuracy while preventing overfitting. LightGBM, another gradient boosting framework, employed histogram-based algorithms and leaf-wise tree growth, allowing faster training on large-scale data (28). The multilayer perceptron (MLP) is a feed-forward neural network composed of input, hidden, and output layers, using nonlinear activation and dropout techniques to capture complex nonlinear relationships between features and outcomes (29). Since different models demonstrate distinct advantages in feature optimization, this study employed DT, RF, XGBoost, LightGBM, and MLP for comparative analysis to evaluate the performance of various machine learning methods. All analyses were conducted in python (version 3.12) using scikit-learn (version1.5.2) for DT, RF, XGBoost (version3.0.4) and LightGBM (version4.6.0). All remaining continuous variables were standardized using z-score normalization. The outcome variable (group) was treated as a binary label. The dataset was randomly split into a training set (70%) and a test set (30%), using stratified sampling by diagnostic group to maintain class balance, with gender and age incorporated as covariates in the model. Additionally, to enhance model usability, feature selection was performed using LASSO regression. LASSO (30) applies L1 regularization to shrink or eliminate less important feature coefficients, reducing model complexity and improving prediction robustness.

### 2.5 Model performance assessment and interpretation

We applied machine learning methods to compare the ADHD group, the s-ADHD group, and the HC group, conducting pairwise analyses for ADHD vs HC, s-ADHD vs HC, and ADHD vs s-ADHD. Hyperparameters for all five models were tuned using a combination of cross-validation (CV) and manual adjustment. Five-fold CV was ultimately applied, with the area under the receiver operating characteristic curve (ROC) used as the primary performance metric to determine the optimal model configuration. In addition to area under the curve (AUC), we evaluated model performance using calibration and classification metrics, including sensitivity, specificity, positive predictive value (PPV), negative predictive value (NPV), accuracy (ACC), F1 score (2×Precision×Recall​/(Precision+Recall)), and Youden’s J index (Sensitivity+Specificity−1). Confusion matrices were generated to visualize the distribution of true positives, false positives, true negatives, and false negatives. Precision-recall curves (PRC) and the corresponding area under the curve (PR-AUC) were also calculated to further assess discriminative ability.

Due to the black-box nature of machine learning models, the contribution of individual features is often difficult to interpret. We used SHAP (31) (version 0.48.0) in python to evaluate feature importance. SHAP employs a game-theoretic approach to aggregate local contributions of features, providing a global explanation of the model and enhancing the interpretability of tree-based models.

## 3 Results

### 3.1 Patient characteristics

We analyzed demographic factors (age, sex), biochemical and routine blood indices (**Supplementary Table S1**), body composition (BMI, PBF) and composite indicators (NLR, PLR, RPR, SII) in 3,678 children with ADHD, 1,495 with s-ADHD and 3,425 HC. The healthy control group had a mean age of 7.73 ± 2.74 years (59.6% males), the s-ADHD group had a mean age of 9.19 ± 2.18 years (78.1% males), and the ADHD group had a mean age of 8.75 ± 1.93 years (81.6% males). All variables followed a normal distribution, as verified through scatter plots. The missing rate for each variable was less than 20%, and multiple imputation was applied to handle missing values. One-way ANOVA followed by LSD *post hoc* tests was performed. Significant group differences were observed across multiple parameters, with distinct trends across the three groups (**Table 1**). ANOVA results revealed significant differences in most parameters among the three groups. Subsequent LSD *post-hoc* tests indicated that both ADHD and s-ADHD groups differed significantly from HC in most parameters, while differences between ADHD and s-ADHD were less pronounced.

**Table 1:** Characteristics of biochemical and routine blood indices in children with diagnosed ADHD, children with subthreshold ADHD, and healthy children.

	Mean ± SD			*F*	*P* ^*^	*P* ^†^	*P* ^†^	*P* ^†^
	Healthy	s - ADHD	ADHD			ADHD vs HC	s - ADHD vs HC	s - ADHD vs ADHD
	n=3425	n=1495	n=3678					
Lipid metabolism								
TP (g/L)	71.00 ± 3.72	71.40 ± 3.51	71.24 ± 3.75	7.32	**0.001**	**0.006**	**<0.001**	0.158
Alb (g/L)	46.16 ± 2.41	45.31 ± 2.29	45.17 ± 2.34	170.21	**<0.001**	**<0.001**	**<0.001**	0.052
Glb (g/L)	24.83 ± 3.31	26.17 ± 3.11	26.04 ± 3.21	154.58	**<0.001**	**<0.001**	**<0.001**	0.181
A/G	1.89 ± 0.29	1.76 ± 0.25	1.76 ± 0.25	245.56	**<0.001**	**<0.001**	**<0.001**	0.968
PA (g/L)	0.21 ± 0.04	0.22 ± 0.04	0.22 ± 0.04	53.56	**<0.001**	**<0.001**	**<0.001**	**<0.001**
K (mmol/L)	4.37 ± 0.31	4.32 ± 0.36	4.34 ± 0.40	10.21	**<0.001**	**0.005**	**<0.001**	**0.038**
Ca (mmol/L)	2.50 ± 0.10	2.56 ± 0.10	2.55 ± 0.10	292.53	**<0.001**	**<0.001**	**<0.001**	**0.034**
Mg (mmol/L)	0.88 ± 0.06	0.92 ± 0.06	0.92 ± 0.09	223.44	**<0.001**	**<0.001**	**<0.001**	0.580
P (mmol/L)	1.59 ± 0.14	1.58 ± 0.16	1.60 ± 0.16	8.42	**<0.001**	**0.001**	0.266	**0.001**
Glu (mmol/L)	4.89 ± 0.41	5.16 ± 0.42	5.12 ± 0.42	349.29	**<0.001**	**<0.001**	**<0.001**	**0.010**
TG (mmol/L)	0.80 ± 0.39	0.86 ± 0.48	0.92 ± 0.61	52.48	**<0.001**	**<0.001**	**<0.001**	**<0.001**
TC (mmol/L)	4.21 ± 0.73	4.21 ± 0.76	4.17 ± 0.72	4.13	**0.016**	**0.006**	0.771	0.068
HDL-C (mmol/L)	1.62 ± 0.33	1.58 ± 0.33	1.55 ± 0.32	32.79	**<0.001**	**<0.001**	**<0.001**	**0.030**
Blood routine inflammation indicators								
WBC (10^9^/L)	7.59 ± 2.58	7.19 ± 1.92	7.39 ± 2.04	17.29	**<0.001**	**<0.001**	**<0.001**	**0.002**
NEUT (10^9^/L)	3.62 ± 1.56	3.44 ± 1.36	3.57 ± 1.45	7.69	**<0.001**	0.161	**<0.001**	**0.003**
LYMPH (10^9^/L)	3.43 ± 1.16	3.06 ± 0.88	3.10 ± 0.89	120.71	**<0.001**	**<0.001**	**<0.001**	0.140
MONO (10^9^/L)	0.53 ± 0.26	0.49 ± 0.17	0.51 ± 0.19	15.1	**<0.001**	**<0.001**	**<0.001**	**0.022**
EO (10^9^/L)	0.33 ± 0.30	0.24 ± 0.21	0.25 ± 0.26	97.95	**<0.001**	**<0.001**	**<0.001**	0.493
BASO (10^9^/L)	0.04 ± 0.03	0.04 ± 0.02	0.04 ± 0.02	0.59	0.553	0.490	0.319	0.583
RBC (10^12^/L)	4.67 ± 0.28	4.72 ± 0.34	4.69 ± 0.34	12.93	**<0.001**	**0.001**	**<0.001**	**0.035**
HGB (g/L)	130.69 ± 9.46	132.11 ± 9.18	130.99 ± 8.75	12.94	**<0.001**	0.168	**<0.001**	**<0.001**
HCT (%)	39.40 ± 2.64	39.79 ± 2.76	39.46 ± 2.71	11.52	**<0.001**	0.347	**<0.001**	**<0.001**
MCV (fL)	84.68 ± 7.45	84.57 ± 4.18	84.09 ± 4.42	9.98	**<0.001**	**<0.001**	0.591	**<0.001**
MCH (pg)	28.14 ± 2.57	28.08 ± 1.65	27.88 ± 1.81	15.00	**<0.001**	**<0.001**	0.400	**<0.001**
MCHC (g/L)	331.52 ± 16.27	332.45 ± 11.10	332.31 ± 12.07	3.84	**0.022**	**0.019**	**0.043**	0.702
RDWSD (fL)	39.67 ± 3.82	38.74 ± 2.51	38.80 ± 2.75	80.45	**<0.001**	**<0.001**	**<0.001**	0.422
RDWCV (fL)	12.87 ± 1.02	12.58 ± 0.75	12.69 ± 0.85	64.71	**<0.001**	**<0.001**	**<0.001**	**<0.001**
PLT (10^9^/L)	288.08 ± 60.25	294.16 ± 62.24	295.19 ± 62.80	12.73	**<0.001**	**<0.001**	**0.001**	0.591
PCT (%)	0.27 ± 0.05	0.28 ± 0.05	0.28 ± 0.05	43.76	**<0.001**	**<0.001**	**<0.001**	0.533
MPV (fL)	9.55 ± 0.92	9.71 ± 0.97	9.73 ± 1.01	34.52	**<0.001**	**<0.001**	**<0.001**	0.634
PDW (fL)	12.88 ± 2.67	11.70 ± 2.61	11.95 ± 2.76	147.57	**<0.001**	**<0.001**	**<0.001**	**0.003**
PLCR (%)	22.00 ± 6.39	22.81 ± 7.43	22.96 ± 7.73	17.28	**<0.001**	**<0.001**	**<0.001**	0.507
Composite indices of systemic inflammation								
NLR	1.11 ± 0.55	1.21 ± 0.83	1.22 ± 0.62	30.47	**<0.001**	**<0.001**	**<0.001**	0.608
PLR	93.09 ± 34.94	103.72 ± 36.53	101.93 ± 32.76	78.06	**<0.001**	**<0.001**	**<0.001**	0.085
RPR	0.05 ± 0.01	0.04 ± 0.01	0.05 ± 0.01	23.26	**<0.001**	**<0.001**	**<0.001**	0.251
SII	318.55 ± 168.02	356.34 ± 247.03	362.46 ± 203.94	46.66	**<0.001**	**<0.001**	**<0.001**	0.358
Body composition								
BMI (kg/m²)	16.45 ± 3.04	17.38 ± 3.16	17.45 ± 3.39	95.95	**<0.001**	**<0.001**	**<0.001**	0.480
PBF (%)	17.97 ± 7.79	19.08 ± 8.30	19.83 ± 8.96	43.53	**<0.001**	**<0.001**	**<0.001**	**0.005**

ADHD, Attention Deficit/Hyperactivity Disorder: Children diagnosed with ADHD according to DSM-5 criteria; s – ADHD, subthreshold ADHD, Children exhibiting symptoms such as impulsivity and hyperactivity, but not meeting the diagnostic criteria for ADHD; TP, Total protein; Alb, Albumin; Glb, Globulin; A/G, Albumin/Globulin ratio; PA, Prealbumin; K, Potassium; Ca, Calcium; Mg, Magnesium; P, Phosphorus; Glu, Glucose; TG, Triglycerides;TC, Total cholesterol; HDL-C, High-density lipoprotein cholesterol; WBC, White blood cell count; NEUT, Neutrophils; LYMPH, Lymphocytes – LYMPH; Mono, Monocytes; EO, ; BASO, Basophils; RBC, Red blood cell count; HGB, Hemoglobin; HCT, Hematocrit; MCV, Mean corpuscular volume; MCH, Mean corpuscular hemoglobin; MCHC, Mean corpuscular hemoglobin concentration; RDW-CV, RDW-SD, Red cell distribution width (coefficient of variation, standard deviation); PLT, Platelet count; PLT, Platelet count; PCT, Plateletcrit; MPV, Mean platelet volume; PDW, Platelet distribution width; PLCR, Platelet large cell ratio; NLR, Neutrophil-to-lymphocyte ratio; PLR, Platelet-to-lymphocyte ratio; RPR, Red cell distribution width-to-platelet ratio; SII, Systemic immune-inflammation index; BMI, Body mass index; PBF, Percentage body fat. *P* < 0.05 was considered statistically significant. Bold highlights *P* < 0.05. P^*^: One-way ANOVA; *P*^†^: LSD Bold indicates

For inflammatory indicators, ADHD and s-ADHD groups exhibited significantly lower lymphocyte counts (LYMPH) compared to HC (F = 120.71, *P* < 0.001), while inflammation-related indices (NLR, PLR, SII) were elevated in both ADHD and s-ADHD groups relative to HC (all *P* < 0.001). For example, SII was higher in ADHD (362.46 ± 203.94) and s-ADHD (356.34 ± 247.03) compared to HC (318.55 ± 168.02). However, no significant differences were found between ADHD and s-ADHD (all *P*>0.05).For protein metabolism indicators in biochemical indices, intergroup differences in total protein (TP), albumin (Alb), globulin (Glb), albumin/globulin ratio (A/G), and prealbumin (PA) were highly significant (all *P* < 0.05). Compared with the HC group, both ADHD and s-ADHD groups showed lower levels of Alb (46.16 ± 2.41, 45.17 ± 2.34, 45.31 ± 2.29 g/L, F = 170.21, *P* < 0.001) and A/G ratio, but higher levels of TP, Glb, and PA. In addition, ADHD and s-ADHD groups had higher glucose (Glu) concentrations than HC (*P* < 0.001). Lipid metabolism indicators revealed elevated triglycerides (TG) and reduced HDL-C levels in both ADHD and s-ADHD groups compared to HC (all *P* < 0.001). Similarly, BMI and PBF were significantly higher in ADHD and s-ADHD groups compared to HC (F = 95.95 and F = 43.53, respectively, both *P* < 0.001).

### 3.2 ML models establishment and comparison

**Figure 1** illustrates the overall workflow for ML model construction and selection: ①Data preparation. After removing outliers, imputing missing values using random imputation, and z-score normalization, the dataset was divided into training and test sets in a 7:3 ratio using stratified sampling. ②Model training. Five machine learning models (DT, RF, XGBoost, LightGBM, and MLP) were trained for three pairwise classifications: ADHD vs HC, ADHD vs s-ADHD, and s-ADHD vs HC. ③Model evaluation. Model performance was assessed on the test set using multiple metrics, including AUC, accuracy, sensitivity, specificity, PPV, NPV, F1 score, and Youden’s J. ROC and precision-recall curves were plotted for visual comparison. Confusion matrices were generated for each model and the classification threshold was examined. ④Model usability. In the three group comparisons, LightGBM achieved the best performance in distinguishing ADHD from healthy controls. To enhance the model’s usability, LASSO regression was applied to select features from 36 candidates, and 11 stable features were retained under the λ.1se criterion. LightGBM models built with 36 vs 11 variables were then compared in terms of ROC-AUC performance. ⑤Model interpretability. Feature contributions for the selected LightGBM model were evaluated using SHAP, and beeswarm plots were generated to rank and visualize important features.

**Figure 2:**
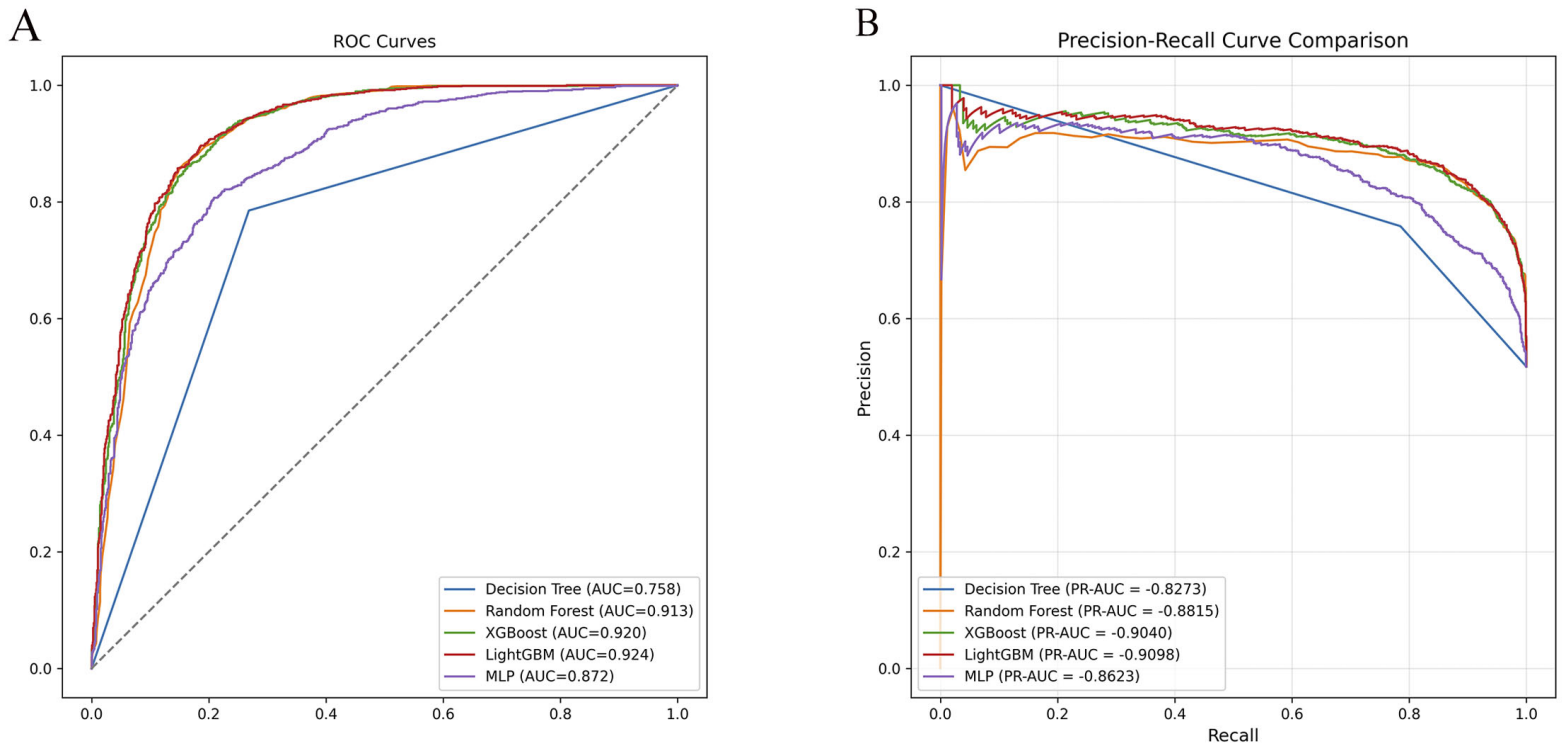
Receiver operating characteristic (ROC) and precision–recall (PR) curve comparisons for ADHD vs healthy controls. **(A)** ROC curves of five machine learning models (DT, RF, XGBoost, LightGBM, MLP) showing model discrimination performance. LightGBM achieved the highest AUC (0.924), followed by XGBoost (0.920) and Random Forest (0.913). **(B)** PR curves demonstrated consistent results, with LightGBM exhibiting the highest PR-AUC (0.9098), confirming its superior classification ability.

**Figure 3:**
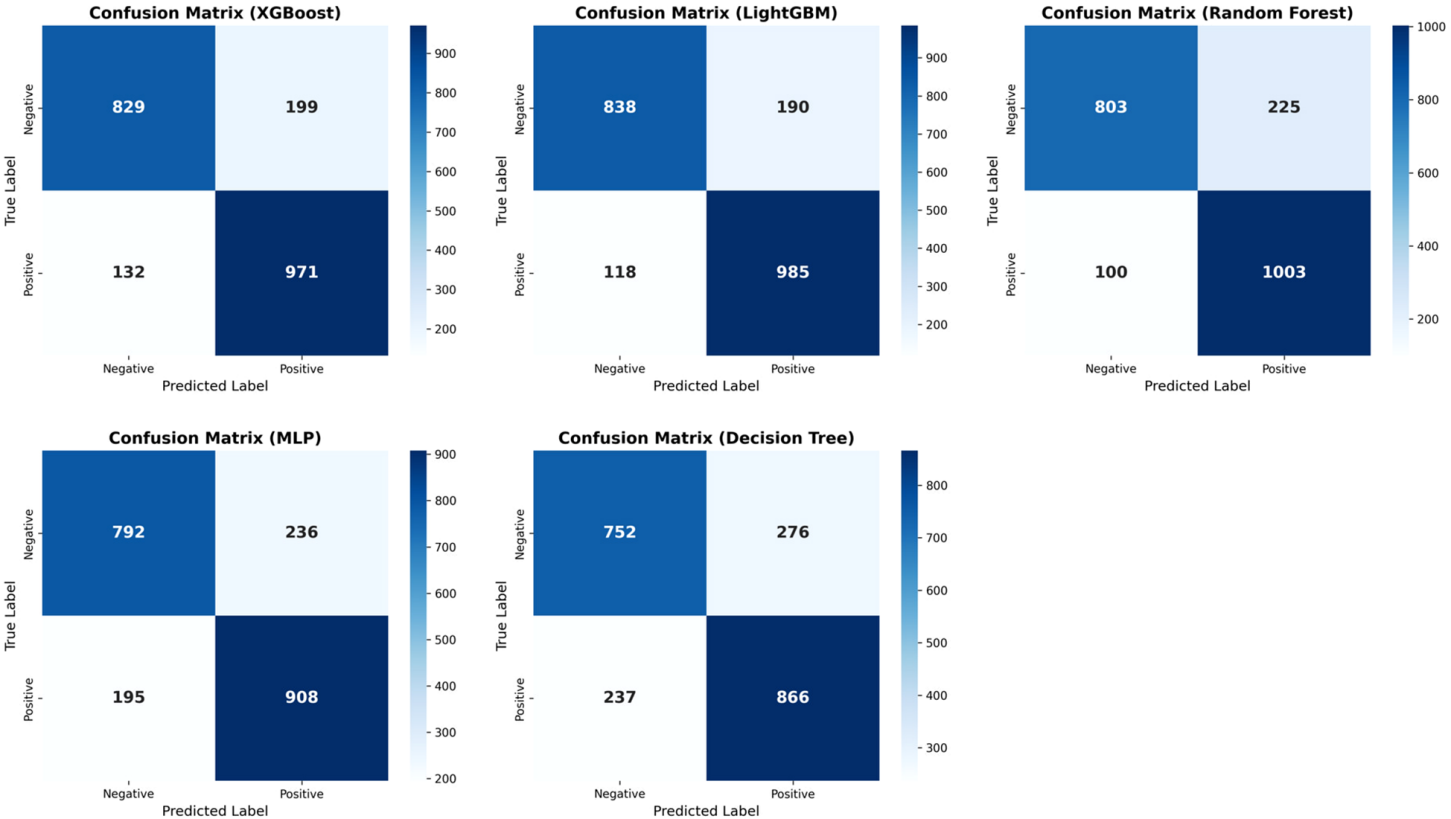
Confusion matrices of five machine learning models for ADHD vs. healthy controls. Confusion matrices display model classification results on the test set. LightGBM achieved the highest accuracy and optimal balance between sensitivity and specificity, followed by Random Forest and XGBoost, indicating its superior performance in distinguishing ADHD from healthy controls.

Across all models and metrics, LightGBM consistently achieved the best performance (**Table 2**). In the ADHD vs HC task, it reached an AUC of 0.924, outperforming XGBoost (0.920) and RF (0.913). Its precision–recall curve also yielded the highest PR-AUC (0.910), confirming robust discriminative power (**Figure 2**). LightGBM also excelled in sensitivity (0.893), specificity (0.815), PPV (0.838), NPV (0.877), accuracy (0.855), and F1 score (0.865). These results demonstrate its balanced ability to identify both positive and negative samples, while maintaining high overall classification accuracy. XGBoost and Random Forest also showed competitive performance, albeit slightly inferior to LightGBM. MLP yielded moderate results, whereas Decision Tree had the weakest performance across most indices (**Table 2**). Confusion matrices for each model are displayed in **Figure 3**. In the s-ADHD vs HC comparison, a similar pattern was observed. LightGBM again achieved the highest AUC (0.914), with XGBoost (0.909) and Random Forest (0.900) performing comparably well, while MLP and Decision Tree underperformed (**Supplementary Figure 1**). In contrast, none of the five models demonstrated meaningful predictive ability in distinguishing ADHD from s-ADHD, with AUCs ranging from 0.529 to 0.674 (**Table 2**), indicating limited predictive capability for this contrast.

**Table 2:** Evaluation of model performance in the three comparison groups using five machine learning models.

	AUC	Sensitivity	Specificity	PPV	NPV	ACC	YoudenJ	F1
ADHD vs HC, 36 variables								
DT	0.758	0.785	0.732	0.758	0.76	0.759	0.517	0.771
RF	0.913	0.909	0.781	0.817	0.889	0.847	0.690	0.861
XGBoost	0.920	0.880	0.806	0.830	0.863	0.845	0.687	0.854
LightGBM	0.924	0.893	0.815	0.838	0.877	0.855	0.708	0.865
MLP	0.872	0.813	0.779	0.798	0.795	0.797	0.592	0.806
ADHD vs HC, 11 variables after LASSO								
DT	0.722	0.740	0.703	0.728	0.716	0.722	0.443	0.734
RF	0.878	0.836	0.781	0.804	0.816	0.809	0.617	0.820
XGBoost	0.877	0.850	0.758	0.790	0.824	0.805	0.607	0.819
LightGBM	0.885	0.858	0.767	0.798	0.834	0.814	0.624	0.827
MLP	0.837	0.802	0.725	0.758	0.774	0.765	0.527	0.779
s-ADHD vs HC, 36 variables								
DT	0.718	0.638	0.797	0.578	0.835	0.749	0.435	0.607
RF	0.900	0.679	0.914	0.776	0.867	0.843	0.593	0.724
XGBoost	0.909	0.741	0.896	0.756	0.888	0.849	0.637	0.749
LightGBM	0.914	0.777	0.898	0.768	0.902	0.861	0.675	0.772
MLP	0.865	0.650	0.900	0.739	0.855	0.824	0.549	0.691
s-ADHD vs HC, 12 variables after LASSO								
DT	0.721	0.605	0.838	0.619	0.829	0.767	0.442	0.612
RF	0.884	0.656	0.910	0.760	0.859	0.833	0.566	0.704
XGBoost	0.883	0.654	0.889	0.720	0.855	0.818	0.543	0.685
LightGBM	0.898	0.703	0.900	0.754	0.874	0.840	0.603	0.727
MLP	0.85	0.592	0.910	0.740	0.836	0.813	0.501	0.658
ADHD vs s-ADHD, 36 variables								
DT	0.529	0.334	0.723	0.330	0.727	0.611	0.058	0.332
RF	0.649	0.087	0.988	0.750	0.727	0.727	0.075	0.156
XGBoost	0.657	0.261	0.916	0.557	0.753	0.726	0.176	0.355
LightGBM	0.674	0.207	0.945	0.604	0.745	0.731	0.152	0.308
MLP	0.608	0.002	1.000	1.000	0.711	0.711	0.002	0.004

ADHD, Attention Deficit/Hyperactivity Disorder: Children diagnosed with ADHD according to DSM-5 criteria; s - ADHD, Children exhibiting symptoms such as impulsivity and hyperactivity, but not meeting the diagnostic criteria for ADHD; AUC, area under the curve; PPV, positive predictive value; NPV, negative predictive value; ACC, Accuracy; F1, 2×Precision×Recall/(Precision+Recall); DT, Decision Tree; RF, Random Forest; MLP, Multilayer Perceptron; XGBoost, Extreme Gradient Boostin; LightGBM, Light Gradient Boosting Machine. ADHD vs HC,11 variables after LASSO: Age, Sex, Alb, A/G, Glu, Ca, Mg, EO, LYMPH, RDWSD, BMI (λ.1se. = 0.038). s- ADHD vs HC, 12 variables after LASSO: Age, Sex, Alb, A/G, Glu, Ca, Mg, EO, LYMPH, PDW, RDWCV, RDWSD (λ.1se. = 0.038).

### 3.3 Model selection and interpretation

**Figure 4:**
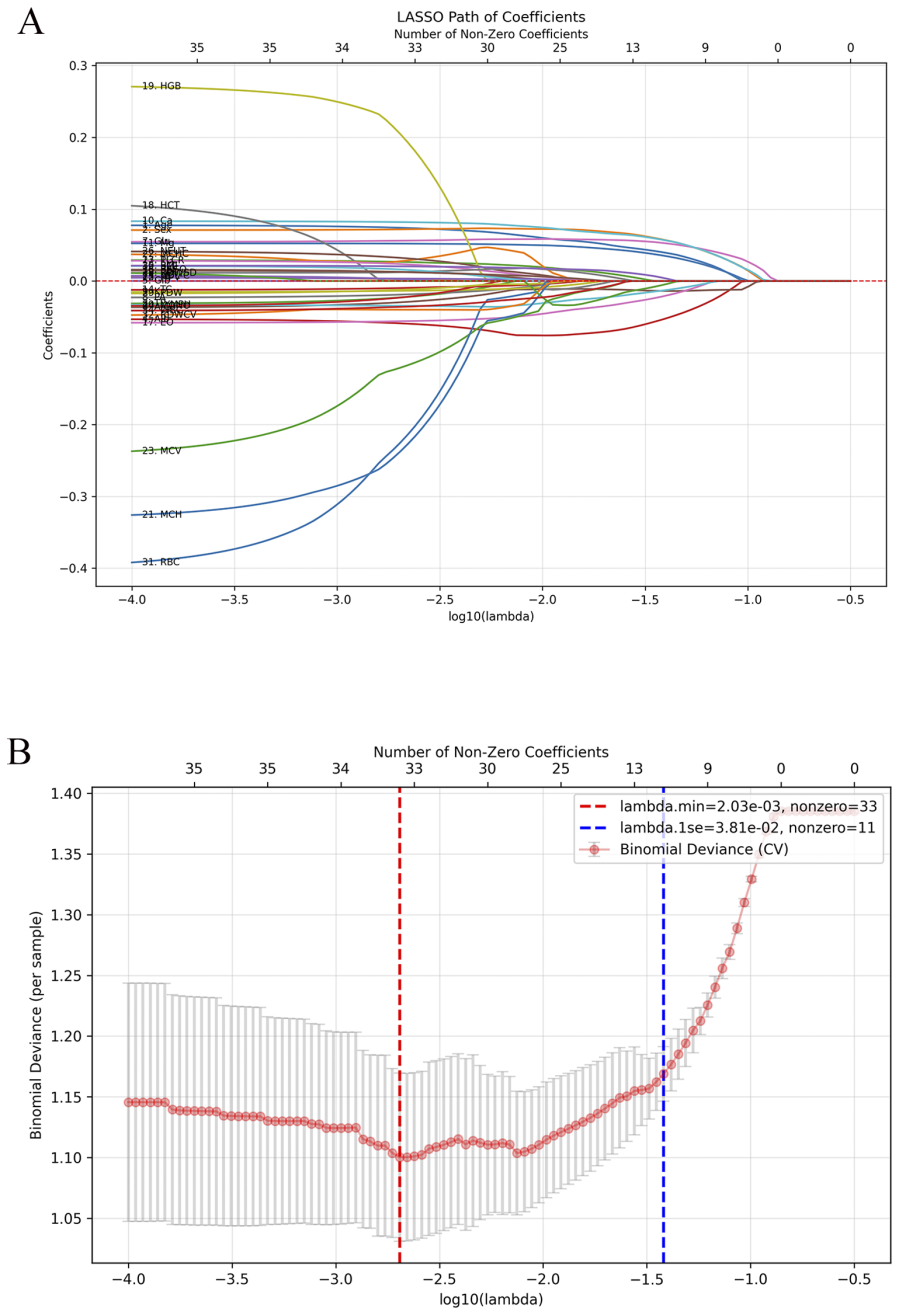
Feature selection using LASSO regression for ADHD vs healthy controls. **(A)** LASSO coefficient paths showing the shrinkage of variables with increasing regularization strength (λ). **(B)** Five-fold cross-validation curve for binomial deviance with λ.min and λ.1se indicated by red and blue dashed lines. At λ.1se (0.038), 11 stable predictors were retained: age, sex, Alb, A/G, Glu, Ca, Mg, EO, LYMPH, RDW-SD, and BMI.

To enhance interpretability and minimize redundancy, LASSO regression was applied with fivefold cross-validation, identifying 11 stable predictors (λ.1se = 0.038). In the ADHD vs HC comparison (**Figure 4**), 11 features were retained at λ.1se (0.038): age (0.0402), sex (0.0579), Alb (−0.0539), A/G (−0.0138), Glu (0.0531), Ca (0.0576), Mg (0.0357), EO (−0.0217), LYMPH (−0.0135), RDW-SD (−0.0058), and BMI (0.0047). Sex, Ca, Glu, and Age had the largest positive coefficients, whereas Alb showed the largest negative coefficient, indicating their predominant contributions to the penalized model. In the s-ADHD vs HC comparison (**Supplementary Figure 2**), 12 nonzero features were retained at λ.1se (0.038): Age, Sex, Alb, A/G, Glu, Ca, Mg, EO, LYMPH, PDW, RDW-CV, and RDW-SD. These variables showed substantial overlap with those identified in the ADHD vs HC model, suggesting a largely consistent set of discriminative features across both comparisons. Performance comparison showed that the reduced 11-variable LightGBM model (AUC = 0.885) retained nearly the same accuracy as the full 36-variable model (AUC = 0.924), confirming the parsimony and robustness of the selected predictors (**Table 2**).

**Figure 5:**
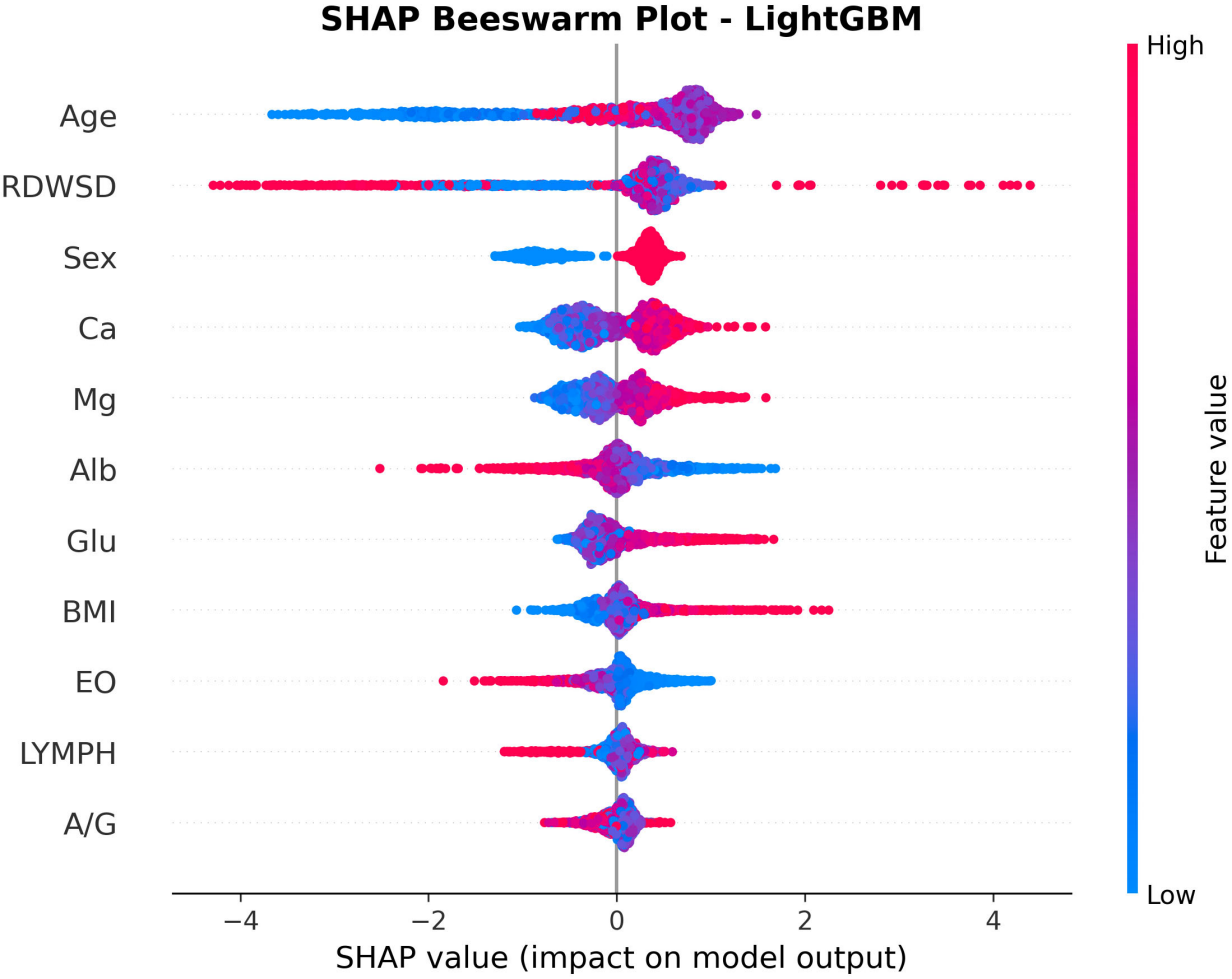
SHAP beeswarm plot for feature contributions in the LightGBM model for ADHD vs healthy controls. The plot shows the impact of 11 selected features on ADHD prediction. Each point represents a sample, with color indicating feature value (red: high; blue: low). Age, RDW-SD, Glu, Ca, and male sex increased ADHD risk, whereas lower Alb and LYMPH values reduced predicted probability, highlighting key contributors to model decision-making.

**Figure 5** presents a SHAP beeswarm plot illustrating the contribution of each feature to the LightGBM model in the ADHD vs HC comparison after LASSO regression. SHAP values are color coded, with blue indicating lower feature values and red indicating higher values, thereby reflecting the relationship between feature levels and prediction outcomes. Age, sex, calcium, and glucose had the strongest positive effects on ADHD prediction, whereas albumin and lymphocytes were negatively associated. The consistency of these markers across both ADHD and s-ADHD comparisons suggests shared biological profiles that distinguish both clinical groups from healthy peers.

## 4 Discussion

ADHD is highly prevalent and imposes substantial burdens on academic performance, daily functioning, and social interactions in affected children (32). However, the etiology of ADHD remains unresolved, and its diagnosis continues to rely predominantly on clinical evaluation, with limited objective laboratory support. In our large-sample study including individuals with ADHD, s-ADHD and HC, we systematically assessed demographic characteristics, routine blood parameters, biochemical indices, and body composition data to delineate their profiles. Moreover, by integrating multiple ML models with LASSO-based feature selection, we aimed to achieve early identification of ADHD, potentially providing a clinically feasible and cost-effective framework to support diagnostic decision-making.

Morphology-derived indices calculated from routine hematological parameters, such as NLR, PLR, RPR, and SII, are widely regarded as objective peripheral markers of systemic inflammatory response. Multiple studies have reported associations between systemic inflammatory alterations and ADHD. For instance, a study from Turkey (12) reported significantly elevated NLR and PLR in children with ADHD compared with controls, with NLR levels unaffected by age, sex, or medication use. Similarly, a meta-analysis (33) from Poland confirmed increased NLR and PLR in ADHD. Interventional studies further indicate that pharmacological treatment may ameliorate inflammatory abnormalities. Atomoxetine (34) has been shown to increase lymphocyte counts and albumin levels, while methylphenidate (35) significantly reduced SII. Consistent with these findings, our study demonstrated significantly higher NLR, PLR, and particularly SII in both ADHD and s-ADHD. Importantly, such abnormalities are not unique to ADHD. Similar evidence has been reported in bipolar disorder(BD) (36) and major depressive disorder (MMD) states (37). These findings indicate that altered peripheral inflammatory profiles are commonly observed across ADHD and other neuropsychiatric conditions.

Routine blood counts and biochemical parameters provide essential information on systemic inflammation, hepatic and renal function, electrolyte balance, and lipid metabolism. Leveraging the diagnostic advantages of machine learning, models built on hematological and biochemical data have been extensively applied to cardiovascular diseases (17), chronic kidney disease (38), sepsis (39) and early tumor detection (40). Based on these considerations, our study incorporated five commonly used machine learning models: DT, RF, MLP, XGBoost and LightGBM. Among these, LightGBM achieved the most favorable balance between predictive accuracy and computational efficiency. This finding is consistent with a study from Jiangnan University (41), which analyzed 242 controls and 498 ADHD cases and identified Gradient Boosting Machine (GBM) as the optimal model. This advantage can be attributed to the characteristics of the present dataset, which features high dimensionality, a mixture of continuous and categorical variables, unequal group sizes, and potential multicollinearity among predictors. LightGBM is particularly well suited for handling such heterogeneous and imbalanced clinical data. Its histogram-based gradient boosting framework enables efficient processing of high-dimensional features while reducing computational complexity, and its built-in categorical feature encoding eliminates the need for one-hot encoding, thereby simplifying data preprocessing. Moreover, the gradient boosting strategy dynamically adjusts feature weights during model training, effectively mitigating the impact of multicollinearity.

In contrast, none of the models showed meaningful discriminatory power between ADHD and s-ADHD. Several possible explanations may account for this observation. First, ADHD and s-ADHD may share substantial biological overlap, as both groups exhibit similar behavioral manifestations of inattention, hyperactivity, and impulsivity. Second, diagnostic ambiguity may contribute, given that ADHD diagnosis primarily relies on behavioral symptoms and the clinical judgment of physicians, which can introduce variability. Third, the hematological and biochemical indicators included in this study may have inherent measurement limitations and insufficient sensitivity to detect subtle metabolic or neurobiological distinctions between the two groups. Finally, algorithmic constraints should also be acknowledged. These factors collectively highlight the complexity of ADHD recognition and diagnosis, emphasizing the need for future research integrating multimodal data to enhance diagnostic precision and model interpretability.

LASSO feature selection substantially reduced the number of variables while maintaining comparable predictive performance in the LightGBM model, thereby enhancing clinical practicality without sacrificing accuracy. The substantial overlap of LASSO-selected variables between the ADHD vs HC and s-ADHD vs HC comparisons, including age, sex, Alb, A/G, Glu, Ca, Mg, EO, LYMPH, and RDW-SD. Notably, our results partially align with those of Xinyu Zhang (41), who identified RDW-SD and EO percentage as key predictors in an ML-based ADHD diagnostic model. These findings demonstrate the robustness and reproducibility of hematology- and biochemistry-based machine learning models for ADHD identification across diverse research settings. Given the black-box nature of machine learning, we applied SHAP analysis to enhance interpretability. SHAP confirmed the importance of several clinical variables, including sex, age, Ca, Glu, and BMI, which contributed strongly to model predictions.

The machine learning model captures data-driven statistical associations, and the findings should be interpreted with caution, taking into account developmental, behavioral, environmental, and methodological factors. In our study, age and sex emerged as two of the most influential predictors in the model. Significant group differences were observed in both variables, with a higher proportion of males and slightly older age in the ADHD group, which is a characteristic feature of real-world data. However, the strong predictive power of these variables likely reflects developmental and epidemiological trends rather than direct mechanistic insights into ADHD pathophysiology. As children age, improvements in executive functioning (42) and increasing behavioral expectations may make ADHD symptoms more detectable and reportable. Similarly, the gender difference is consistent with established epidemiological evidence where the prevalence rate in males is significantly higher than that in females.

Our study found significantly higher BMI and Glu levels in children with ADHD, consistent with previous literature. A Brazilian cohort study (21), showing that higher childhood ADHD scores predicted abnormal body fat composition in early adulthood, possibly via binge eating and impaired emotional regulation. Likewise, a study from Upstate Medical University (43) identified a significant correlation between ADHD symptoms and elevated Glu levels, while research from Thailand (44) further indicated that higher intake of sugar-sweetened beverages was associated with increased ADHD symptom risk among university students. Previous evidence suggests that children with ADHD exhibit nearly twice the prevalence of unhealthy lifestyle behaviors (45) compared with typically developing peers. It is noteworthy that lifestyle factors such as dietary patterns, nutritional intake, and sleep quality play a crucial role in the onset and prognosis of psychiatric disorders (46). Our study also revealed significantly reduced TC and HDL-C in ADHD and s-ADHD, consistent with previous evidence, including findings by Sibelnur Avcil (14) linking low cholesterol to hyperactivity in boys. Such alterations in lipid homeostasis may affect serotonergic signaling via cholesterol-dependent modulation of membrane microdomains (47), potentially contributing to aggression, impulsivity, and ADHD symptoms.

RDW-SD and albumin were identified as core predictive features for analysis in our study. RDW reflects the degree of heterogeneity in red blood cell volume (48), while albumin is the most abundant circulating protein in plasma. Both are widely recognized as comprehensive associative markers associated with inflammatory responses, oxidative stress, and nutritional status. A study from Iran (49) confirmed that RDW levels are significantly elevated in individuals with mental disorders such as depression and anxiety, suggesting that RDW may be associated with neurodevelopmental or emotional regulation processes. However, these associations are more likely influenced by interacting factors such as nutritional or inflammatory states, rather than being specific to any single disease process.

In addition, our study identified serum calcium and magnesium levels as potential predictive indicators of ADHD. Calcium ions (50) play a central role in neuronal membrane depolarization and synaptic plasticity, while magnesium, as a natural NMDA receptor antagonist, modulates calcium influx and participates in regulating neuronal excitability and neuroinflammatory responses. The two elements work synergistically to maintain neurotransmitter release, synaptic homeostasis, and neural signal integration. Previous studies have reported lower serum magnesium levels in individuals with autism spectrum disorder and depression (51). Animal studies have further shown that L-type calcium channel blockers (such as amlodipine) can reduce hyperactivity and impulsive behaviors in rodent and zebrafish models (52), supporting a potential role of calcium signaling pathways in behavioral regulation. However, serum Ca and Mg levels can only indirectly reflect systemic homeostasis, their relationship with intracellular concentrations and neural function requires further elucidation through molecular and neuroimaging studies.

Although the identified inflammatory and metabolic features provided strong discriminative power for our diagnostic model, the cross-sectional design of this study only reveals associations and does not allow determination of whether these markers represent antecedent risk factors or downstream consequences of the disorder. Previous research has shown that irregular sleep patterns (53), disordered eating behaviors (46), and hyperactivity commonly observed in children with ADHD can influence energy metabolism and gut microbiota homeostasis, thereby altering glucose, lipid, and inflammatory marker levels. Moreover, it cannot be excluded that genetic factors (6, 7) or environmental influences may concurrently drive both ADHD symptoms and associative markers alterations. Future studies should adopt longitudinal cohort designs, following children from the preschool period and continuously monitoring inflammatory and metabolic trajectories before and after the onset of ADHD symptoms. Such approaches will help elucidate the temporal relationships underlying these associations and provide more robust evidence for the biological mechanisms of ADHD.

Taken together, our study has several strengths. First, only a few ML models incorporating laboratory indices for ADHD have been reported to date, whereas our study included a large sample of 8,598 children, enhancing the reliability of the findings. Second, the use of routine hematological, biochemical and body composition parameters, which are cost-effective, easily accessible, and highly feasible, offers practical value and generalizability. These measures help to address the lack of objective laboratory evidence in ADHD diagnosis and may reduce the risk of underdiagnosis. Third, we included children with s-ADHD, who present clinical symptoms but do not meet DSM-IV diagnostic criteria, thereby facilitating the early identification of children at potential risk for ADHD. Finally, by integrating multiple machine learning models with ANOVA analysis, LASSO-based feature selection, and SHAP interpretability analysis, our study not only achieved strong predictive performance but also provided robust explanatory value.

This study also has several limitations. First, as a cross-sectional analysis focusing solely on laboratory parameters, it did not account for potential confounding factors such as genetic background, lifestyle, medication use, comorbidities, nutritional status, environmental exposures, and socioeconomic conditions, all of which may influence the interpretability of the model. Second, differences in age and sex distribution were observed among the groups, which are inherent characteristics of real-world data. The ADHD cohort consisted of outpatients who may represent children with more clinically pronounced symptoms, whereas the healthy control group was recruited from physical examination clinics and may come from families with greater health awareness or easier access to preventive healthcare. These characteristics could introduce bias, and therefore, the findings should be interpreted within the context of this specific clinical population. Third, as a single-center study without external validation, our results can only demonstrate associations rather than causal or temporal relationships between ADHD and the identified associative markers. External validation using independent or multicenter datasets is needed to confirm the stability and generalizability of the selected features. Future longitudinal and multicenter studies incorporating multimodal data, including neuroimaging, behavioral assessments, genetic background, and social environmental factors, are warranted to further clarify causal directions and enhance the translational robustness of these findings.

## 5 Conclusion

This study demonstrates that routine clinical indicators, particularly systemic inflammation markers, serum biochemical parameters, routine blood counts, and body composition measures, hold potential as objective associative markers for ADHD. Using Light Gradient Boosting Machine (LightGBM), we established a low-cost and easily applicable model integrating routine blood tests, biochemical indices, body composition, and demographic information, which showed strong performance in differentiating ADHD and subthreshold ADHD from healthy controls. Our findings indicate that associative-marker-driven machine learning models may serve as effective auxiliary tools for early screening and objective diagnosis of ADHD.

## Data Availability

The raw data supporting the conclusions of this article will be made available by the authors, without undue reservation.

## References

[1] Posner J Polanczyk GV Sonuga-Barke E . Attention-deficit hyperactivity disorder. Lancet. (2020) 395:450–62. doi: 10.1016/s0140-6736(19)33004-1, PMID: 31982036 PMC7880081

[2] Ayano G Demelash S Gizachew Y Tsegay L Alati R . The global prevalence of attention deficit hyperactivity disorder in children and adolescents: An umbrella review of meta-analyses. J Affect Disord. (2023) 339:860–6. doi: 10.1016/j.jad.2023.07.071, PMID: 37495084

[3] Di Lorenzo R Balducci J Poppi C Arcolin E Cutino A Ferri P . Children and adolescents with ADHD followed up to adulthood: a systematic review of long-term outcomes. Acta Neuropsychiatr. (2021) 33:283–98. doi: 10.1017/neu.2021.23, PMID: 34384511

[4] Schiweck C Arteaga-Henriquez G Aichholzer M Edwin Thanarajah S Vargas-Cáceres S Matura S . Comorbidity of ADHD and adult bipolar disorder: A systematic review and meta-analysis. Neurosci Biobehav Rev. (2021) 124:100–23. doi: 10.1016/j.neubiorev.2021.01.017, PMID: 33515607

[5] MaChado A Rafaela D Silva T Veigas T Cerejeira J . ADHD among offenders: prevalence and relationship with psychopathic traits. J Attention Disord. (2020) 24:2021–9. doi: 10.1177/1087054717744880, PMID: 29199502

[6] Kian N Samieefar N Rezaei N . Prenatal risk factors and genetic causes of ADHD in children. World J pedia: WJP. (2022) 18:308–19. doi: 10.1007/s12519-022-00524-6, PMID: 35235183

[7] Yadav SK Bhat AA Hashem S Nisar S Kamal M Syed N . Genetic variations influence brain changes in patients with attention-deficit hyperactivity disorder. Trans Psychiatry. (2021) 11:349. doi: 10.1038/s41398-021-01473-w, PMID: 34091591 PMC8179928

[8] Compa M Baumbach C Kaczmarek-Majer K Buczylowska D Gradys GO Skotak K . Air pollution and attention in Polish schoolchildren with and without ADHD. Sci Tot Environ. (2023) 892:164759. doi: 10.1016/j.scitotenv.2023.164759, PMID: 37302611

[9] Brown-Leung JM Cannon JR . Neurotransmission targets of per- and polyfluoroalkyl substance neurotoxicity: mechanisms and potential implications for adverse neurological outcomes. Chem Res Toxicol. (2022) 35(8):1312–1333. Available online at. doi: 10.1021/acs.chemrestox.2c00072, PMID: 35921496 PMC10446502

[10] Dunn GA Nigg JT Sullivan EL . Neuroinflammation as a risk factor for attention deficit hyperactivity disorder. Pharmacol Biochem Behav. (2019) 182:22–34. doi: 10.1016/j.pbb.2019.05.005, PMID: 31103523 PMC6855401

[11] Visternicu M Rarinca V Burlui V Halitchi G Ciobică A Singeap AM . Investigating the impact of nutrition and oxidative stress on attention deficit hyperactivity disorder. Nutrients. (2024) 16(18):3113. doi: 10.3390/nu16183113, PMID: 39339712 PMC11435085

[12] Onder A Gizli Coban O Surer Adanir A . Elevated neutrophil-to-lymphocyte ratio in children and adolescents with attention-deficit/hyperactivity disorder. Int J Psychiatry Clin Pract. (2021) 25:43–8. doi: 10.1080/13651501.2020.1804940, PMID: 32787596

[13] von Eckardstein A Nordestgaard BG Remaley AT Catapano AL . High-density lipoprotein revisited: biological functions and clinical relevance. Eur Heart J. (2023) 44:1394–407. doi: 10.1093/eurheartj/ehac605, PMID: 36337032 PMC10119031

[14] Avcil S . Association between altered lipid profiles and attention deficit hyperactivity disorder in boys. Nordic J Psychiatry. (2018) 72(5):361–366. Available online at. doi: 10.1080/08039488.2018.1465591, PMID: 29688116

[15] Swanson K Wu E Zhang A Alizadeh AA Zou J . From patterns to patients: Advances in clinical machine learning for cancer diagnosis, prognosis, and treatment. Cell. (2023) 186:1772–91. doi: 10.1016/j.cell.2023.01.035, PMID: 36905928

[16] Erickson BJ Korfiatis P Akkus Z Kline TL . Machine learning for medical imaging. Radiograph: A Rev Publ Radiol Soc North Amer Inc. (2017) 37:505–15. doi: 10.1148/rg.2017160130, PMID: 28212054 PMC5375621

[17] Wang Z Gu Y Huang L Liu S Chen Q Yang Y . Construction of machine learning diagnostic models for cardiovascular pan-disease based on blood routine and biochemical detection data. Cardiovasc Diabetol. (2024) 23:351. doi: 10.1186/s12933-024-02439-0, PMID: 39342281 PMC11439295

[18] Handelman GS Kok HK Chandra RV Razavi AH Lee MJ Asadi H . eDoctor: machine learning and the future of medicine. J Internal Med. (2018) 284:603–19. doi: 10.1111/joim.12822, PMID: 30102808

[19] Peterson BS Trampush J Maglione M Bolshakova M Rozelle M Miles J . Treatments for ADHD in children and adolescents: A systematic review. Pediatrics. (2024) 153(4):e2024065787. doi: 10.1542/peds.2024-065787, PMID: 38523592

[20] Pereira-Sanchez V Castellanos FX . Neuroimaging in attention-deficit/hyperactivity disorder. Curr Opin Psychiatry. (2021) 34:105–11. doi: 10.1097/YCO.0000000000000669, PMID: 33278156 PMC7879851

[21] Choi H Hong J Kang HG Park MH Ha S Lee J . Retinal fundus imaging as biomarker for ADHD using machine learning for screening and visual attention stratification. NPJ Digit Med. (2025) 8:164. doi: 10.1038/s41746-025-01547-9, PMID: 40097590 PMC11914053

[22] Loe IM Kakar PA Sanders LM . Diagnosis, evaluation, and treatment of attention-deficit/hyperactivity disorder. JAMA Pediatr. (2021) 175:191–2. doi: 10.1001/jamapediatrics.2020.2218, PMID: 32777021

[23] Avcil S . Evaluation of the neutrophil/lymphocyte ratio, platelet/lymphocyte ratio, and mean platelet volume as inflammatory markers in children with attention-deficit hyperactivity disorder. Psychiatry Clin Neurosci. (2018) 72:522–30. doi: 10.1111/pcn.12659, PMID: 29607599

[24] Austin PC White IR Lee DS van Buuren S . Missing data in clinical research: A tutorial on multiple imputation. Can J Cardiol. (2021) 37:1322–31. doi: 10.1016/j.cjca.2020.11.010, PMID: 33276049 PMC8499698

[25] Luo Y Ding W Yang X Bai H Jiao F Guo Y . Construction and validation of a predictive model for meningoencephalitis in pediatric scrub typhus based on machine learning algorithms. Emerg Microbes Infect. (2025) 14:2469651. doi: 10.1080/22221751.2025.2469651, PMID: 39964062 PMC11892057

[26] Delebarre M Gonzales F Behal H Tiphaine A Sudour-Bonnange H Lutun A . Decision-tree derivation and external validation of a new clinical decision rule (DISCERN-FN) to predict the risk of severe infection during febrile neutropenia in children treated for cancer. Lancet Child Adolesc Health. (2022) 6:260–8. doi: 10.1016/S2352-4642(21)00337-0, PMID: 34871572

[27] Zhou X Zhang J Deng X-M Fu F-M Wang J-M Zhang Z-Y . Using random forest and biomarkers for differentiating COVID-19 and Mycoplasma pneumoniae infections. Sci Rep. (2024) 14:22673. doi: 10.1038/s41598-024-74057-5, PMID: 39349769 PMC11442435

[28] Zhang M Zhao C Cheng Q Xu J Xu N Yu L . A score-based method of immune status evaluation for healthy individuals with complete blood cell counts. BMC Bioinf. (2023) 24:467. doi: 10.1186/s12859-023-05603-7, PMID: 38082403 PMC10714576

[29] Aguirre U Urrechaga E . Diagnostic performance of machine learning models using cell population data for the detection of sepsis: a comparative study. Clin Chem Lab Med. (2023) 61:356–65. doi: 10.1515/cclm-2022-0713, PMID: 36351434

[30] Song B Liu P Fu K Liu C . Developing a predictive model for septic shock risk in acute pancreatitis patients using interpretable machine learning algorithms. Digit Health. (2025) 11:20552076251346361. doi: 10.1177/20552076251346361, PMID: 40433305 PMC12107010

[31] Qi X Wang S Fang C Jia J Lin L Yuan T . Machine learning and SHAP value interpretation for predicting comorbidity of cardiovascular disease and cancer with dietary antioxidants. Redox Biol. (2025) 79:103470. doi: 10.1016/j.redox.2024.103470, PMID: 39700695 PMC11729017

[32] Faraone SV Bellgrove MA Brikell I Cortese S Hartman CA Hollis C . Attention-deficit/hyperactivity disorder. Nat Rev Dis Prim. (2024) 10:11. doi: 10.1038/s41572-024-00495-0, PMID: 38388701

[33] Gedek A Modrzejewski S Gedek M Antosik AZ Mierzejewski P Dominiak M . Neutrophil to lymphocyte ratio, platelet to lymphocyte ratio, and monocyte to lymphocyte ratio in ADHD: a systematic review and meta-analysis. Front Psychiatry. (2023) 14:1258868. doi: 10.3389/fpsyt.2023.1258868, PMID: 38034918 PMC10682201

[34] Öz E Parlak ME Kapıcı Y Balatacı U Küçükkelepçe O Kurt F . Pre- and post-treatment evaluation of routine blood analysis in patients with attention deficit hyperactivity disorder and comparison with the healthy control group. Sci Rep. (2023) 13:16233. doi: 10.1038/s41598-023-43553-5, PMID: 37758832 PMC10533532

[35] Kasak M Gunal Okumus H Celik YS Kirsan FZ Ozturk Y Efe A . Novel hematologic ratios and systemic inflammation index in ADHD: effects of methylphenidate treatment. Front Psychiatry. (2025) 16:1621767. doi: 10.3389/fpsyt.2025.1621767, PMID: 40740259 PMC12308500

[36] Mazza MG Lucchi S Tringali AGM Rossetti A Botti ER Clerici M . Neutrophil/lymphocyte ratio and platelet/lymphocyte ratio in mood disorders: A meta-analysis. Prog Neuropsychopharmacol Biol Psychiatry. (2018) 84(Pt A):229–236. doi: 10.1016/j.pnpbp.2018.03.012, PMID: 29535038

[37] Wei Y Feng J Ma J Chen D Chen J . Neutrophil/lymphocyte, platelet/lymphocyte and monocyte/lymphocyte ratios in patients with affective disorders. J Affect Disord. (2022) 309:221–8. doi: 10.1016/j.jad.2022.04.092, PMID: 35460739

[38] He J Wang X Zhu P Wang X Zhang Y Zhao J . Identification and validation of an explainable early-stage chronic kidney disease prediction model: a multicenter retrospective study. EClinicalMedicine. (2025) 84:103286. doi: 10.1016/j.eclinm.2025.103286, PMID: 40567347 PMC12192353

[39] Steinbach D Ahrens PC Schmidt M Federbusch M Heuft L Lübbert C . Applying machine learning to blood count data predicts sepsis with ICU admission. Clin Chem. (2024) 70:506–15. doi: 10.1093/clinchem/hvae001, PMID: 38431275

[40] Li Z Jiang Y Li B Han Z Shen J Xia Y . Development and validation of a machine learning model for detection and classification of tertiary lymphoid structures in gastrointestinal cancers. JAMA net Open. (2023) 6:e2252553. doi: 10.1001/jamanetworkopen.2022.52553, PMID: 36692877 PMC10408275

[41] Zhang X Xiao X Luo Y Xiao W Cao Y Chang Y . Machine learning based early diagnosis of ADHD with SHAP value interpretation: A retrospective observational study. Neuropsychiatr Dis Treat. (2025) 21:1075–90. doi: 10.2147/NDT.S519492, PMID: 40417187 PMC12103855

[42] Liang X Li R Wong SHS Sum RKW Sit CHP . The impact of exercise interventions concerning executive functions of children and adolescents with attention-deficit/hyperactive disorder: a systematic review and meta-analysis. Int J Behav Nutr Phys Act. (2021) 18:68. doi: 10.1186/s12966-021-01135-6, PMID: 34022908 PMC8141166

[43] Dehnavi AZ Zhang-James Y Draytsel D Carguello B Faraone SV Weinstock RS . Association of ADHD symptoms with type 2 diabetes and cardiovascular comorbidities in adults receiving outpatient diabetes care. J Clin Transl Endocrinol. (2023) 32:100318. doi: 10.1016/j.jcte.2023.100318, PMID: 37124458 PMC10130340

[44] Yingchankul N Panuspanudechdamrong C Techapipatchai N Chanmuang T Netsiri P Karawekpanyawong N . Is the consumption of added sugar from common beverages associated with the presence of attention deficit hyperactivity disorder symptoms in thai medical students? Nutrients. (2023) 15(20):4395. doi: 10.3390/nu15204395, PMID: 37892470 PMC10610093

[45] Holton KF Nigg JT . The association of lifestyle factors and ADHD in children. J Atten Disord. (2020) 24:1511–20. doi: 10.1177/1087054716646452, PMID: 27125993 PMC5205565

[46] Firth J Solmi M Wootton RE Vancampfort D Schuch FB Hoare E . A meta-review of "lifestyle psychiatry": the role of exercise, smoking, diet and sleep in the prevention and treatment of mental disorders. World Psychiatry. (2020) 19:360–80. doi: 10.1002/wps.20773, PMID: 32931092 PMC7491615

[47] Troisi A . Low cholesterol is a risk factor for attentional impulsivity in patients with mood symptoms. Psychiatry Res. (2011) 188:83–7. doi: 10.1016/j.psychres.2010.11.005, PMID: 21112642

[48] Hao M Jiang S Tang J Li X Wang S Li Y . Ratio of red blood cell distribution width to albumin level and risk of mortality. JAMA Netw Open. (2024) 7:e2413213. doi: 10.1001/jamanetworkopen.2024.13213, PMID: 38805227 PMC11134218

[49] Shafiee M Tayefi M Hassanian SM Ghaneifar Z Parizadeh MR Avan A . Depression and anxiety symptoms are associated with white blood cell count and red cell distribution width: A sex-stratified analysis in a population-based study. Psychoneuroendocrinology. (2017) 84:101–8. doi: 10.1016/j.psyneuen.2017.06.021, PMID: 28697416

[50] Ugarte G Piña R Contreras D Godoy F Rubio D Rozas C . Attention deficit-hyperactivity disorder (ADHD): from abnormal behavior to impairment in synaptic plasticity. Biol (Basel). (2023) 12(9):1241. doi: 10.3390/biology12091241, PMID: 37759640 PMC10525904

[51] Klocke B Krone K Tornes J Moore C Ott H Pitychoutis PM . Insights into the role of intracellular calcium signaling in the neurobiology of neurodevelopmental disorders. Front Neurosci. (2023) 17:1093099. doi: 10.3389/fnins.2023.1093099, PMID: 36875674 PMC9975342

[52] Þorsteinsson H Baukmann HA Sveinsdóttir HS Halldórsdóttir D Grzymala B Hillman C . Validation of L-type calcium channel blocker amlodipine as a novel ADHD treatment through cross-species analysis, drug-target Mendelian randomization, and clinical evidence from medical records. Neuropsychopharmacology. (2025) 50:1145–55. doi: 10.1038/s41386-025-02062-x, PMID: 39953207 PMC12089589

[53] Stein MA Weiss MD . Editorial: longitudinal associations between sleep and ADHD symptoms: ADHD is a 24-hour disorder. J Am Acad Child Adolesc Psychiatry. (2023) 62:133–4. doi: 10.1016/j.jaac.2022.11.003, PMID: 36400280

