# Supplementary figures and images for: Machine learning–guided feature selection and predictive model construction for attention-deficit/hyperactivity disorder
Source: Front Psychiatry. 2025 Dec 17;16:1724359. doi: 10.3389/fpsyt.2025.1724359 (PMC12753890; doi:10.3389/fpsyt.2025.1724359)

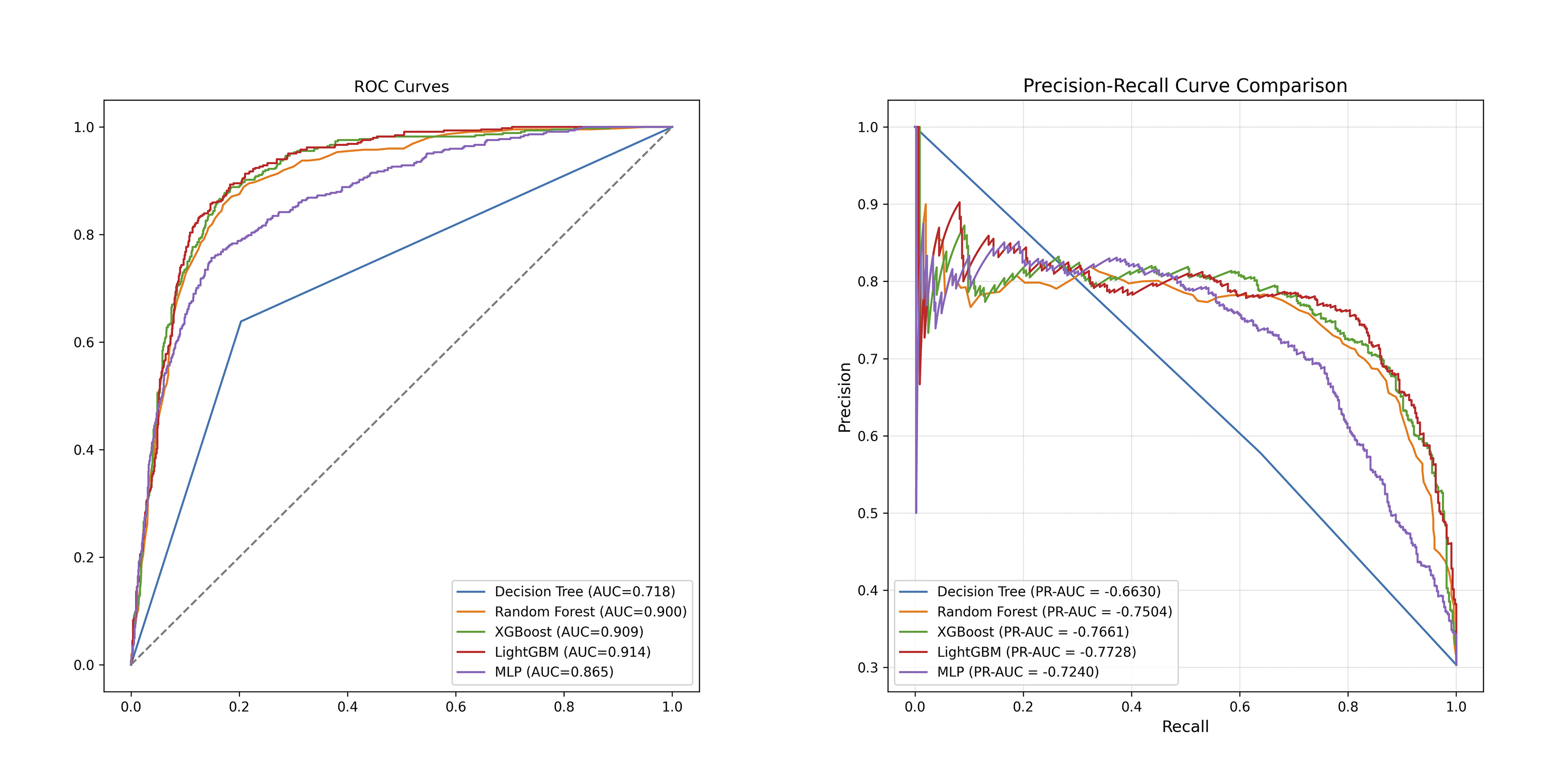

Supplement: Supplementary file 1 [file Image1.tif]

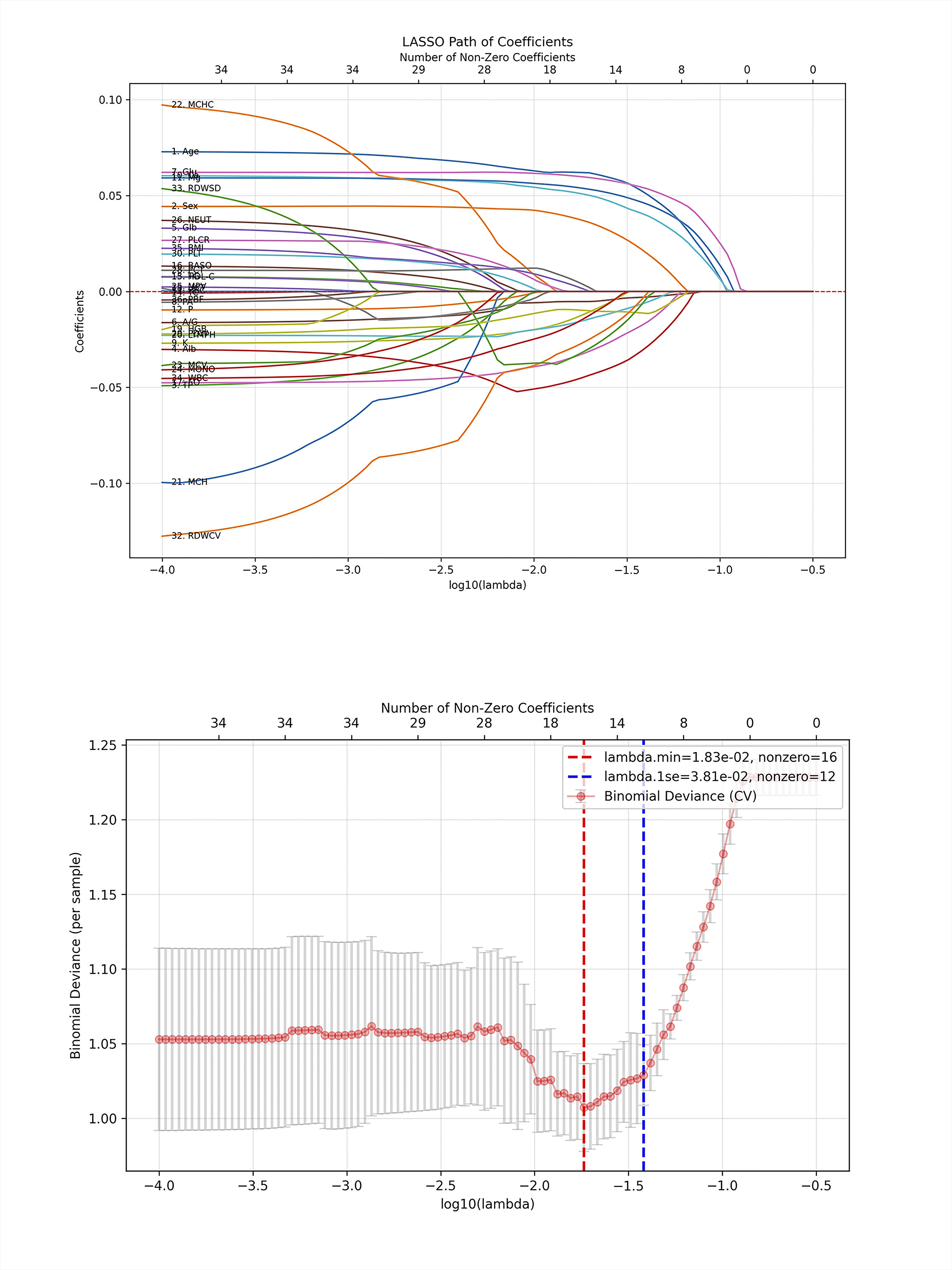

Supplement: Supplementary file 2 [file Image2.tif]
