# Supplementary material for: Machine learning–guided feature selection and predictive model construction for attention-deficit/hyperactivity disorder
Source: Front Psychiatry. 2025 Dec 17;16:1724359. doi: 10.3389/fpsyt.2025.1724359 (PMC12753890; doi:10.3389/fpsyt.2025.1724359)
Supplement: Supplementary file 3 [file Table1.docx]

**Supplementary Table S1. Biochemical and routine blood indices included in the study**

| **Category** | **Indices** | **Abbreviations** |
| --- | --- | --- |
| **Protein metabolism** | Total protein,  Albumin,  Globulin,  Albumin/Globulin ratio,  Prealbumin | TP, Alb, Glb, A/G, PA |
| **Lipid and glucose metabolism** | Triglycerides,  Total cholesterol,  High-density lipoprotein cholesterol,  Glucose | TG, TC, HDL-C, Glu |
| **Electrolytes** | Potassium,  Calcium,  Magnesium,  Phosphorus | K, Ca, Mg, P |
| **Red blood cell parameters** | Red blood cell count,  Hemoglobin,  Hematocrit,  Mean corpuscular volume,  Mean corpuscular hemoglobin,  Mean corpuscular hemoglobin concentration,  Red cell distribution width (coefficient of variation, standard deviation) | RBC, HGB, HCT, MCV, MCH, MCHC, RDW-CV, RDW-SD |
| **Platelet indices** | Platelet count,  Mean platelet volume,  Plateletcrit,  Platelet distribution width,  Platelet large cell ratio | PLT, MPV, PCT, PDW, PLCR |
| **Leukocyte parameters** | White blood cell count,  Basophils,  Eosinophils,  Lymphocytes,  Monocytes,  Neutrophils | WBC, BASO, EO, LYMPH, MONO, NEUT |
